# A Case of Delayed Treatment in Cervical Spondylotic Myelopathy Presenting as Hemiplegia in an Elderly Female

**DOI:** 10.7759/cureus.28776

**Published:** 2022-09-04

**Authors:** Sharon Hechter, Ankita Prasad, Andrea C Marin, Kajal Ghodasara, Sandeep Pavuluri, Zehra Taqvi, Saniya H Patel, Sophia Ji, Pramil Cheriyath

**Affiliations:** 1 Internal Medicine, Hackensack Meridian Health Ocean Medical Center, Brick, USA; 2 Internal Medicine, Ocean University Medical Center, Brick, USA; 3 Research, Hackensack Meridian Health Ocean Medical Center, Brick, USA; 4 Research, Ocean University Medical Center, Brick, USA

**Keywords:** paralysis, neck pain, hemiplegia, paraesthesia, spine, cervical spondylomyelopathy

## Abstract

Cervical spondylotic myelopathy (CSM) is a degenerative disease of the spine that occurs mainly in the elderly, along with age-related changes in the spine. It has a varied presentation, from nonspecific paraesthesia and neck pain to hemiplegia and paraplegia, even quadriplegia, due to the compression of the cervical spine or nerves in the spinal canal or foramina. The diagnosis is often delayed, and cases may present as acute worsening and even hemiplegia mimicking stroke following trauma or neck manipulation. We present a case of CSM in an elderly female presenting with hemiplegia. There was a good recovery after surgical decompression. This case highlights the importance of a high index of suspicion and early diagnosis.

## Introduction

Cervical spondylotic myelopathy (CSM) is a common spinal degenerative disease that causes spinal cord dysfunction, especially in adults aged 55 and older. The associated neurological symptoms can vary from nonspecific neck pain and stiffness, occipital headaches, mild paresthesia, loss of dexterity in hand movements and gait, bowel and bladder habitus, to quadriplegia affecting the quality of life. The temporal presentation may also vary; some rapidly worsen acutely after trauma or neck manipulation, whereas others can present gradually over time with motor weakness. Cervical neck trauma, congenital spinal canal stenosis, and degenerative bone diseases like osteoporosis are risk factors associated with aging and predispose to CSM.

The prevalence of CSM that has been surgically treated is estimated to be 1.6 per 100,000 people [1]. Despite being not uncommon, there is a diagnostic delay in most cases. Worsening symptoms may be present for some time and are wrongly attributed to functional impairment due to aging. The classic triad of symptoms that can help consider CSM as a differential are poor hand dexterity, new unsteady walking patterns, and new-onset and growing problems with motor abilities [2]. Timely treatment of the symptoms can relieve many acute symptoms. Surgical treatment, when indicated, is the definitive treatment. Conservative management helps manage the symptoms. To avoid neurological sequelae, physicians and orthopedic surgeons must have a greater index of suspicion for this condition, as it can help in early detection and management. Cervical myelopathy's natural history includes static and dynamic factors [3]. Static factors such as osteophyte formation and ligamentum flavum hypertrophy can reduce the spinal canal diameter and promote cord compression [3]. Dynamic factors become relevant when the normal motion of the cervical spine causes static components to interact in such a manner that aggravates or promotes spinal cord damage [3].

## Case presentation

**Figure 1:**
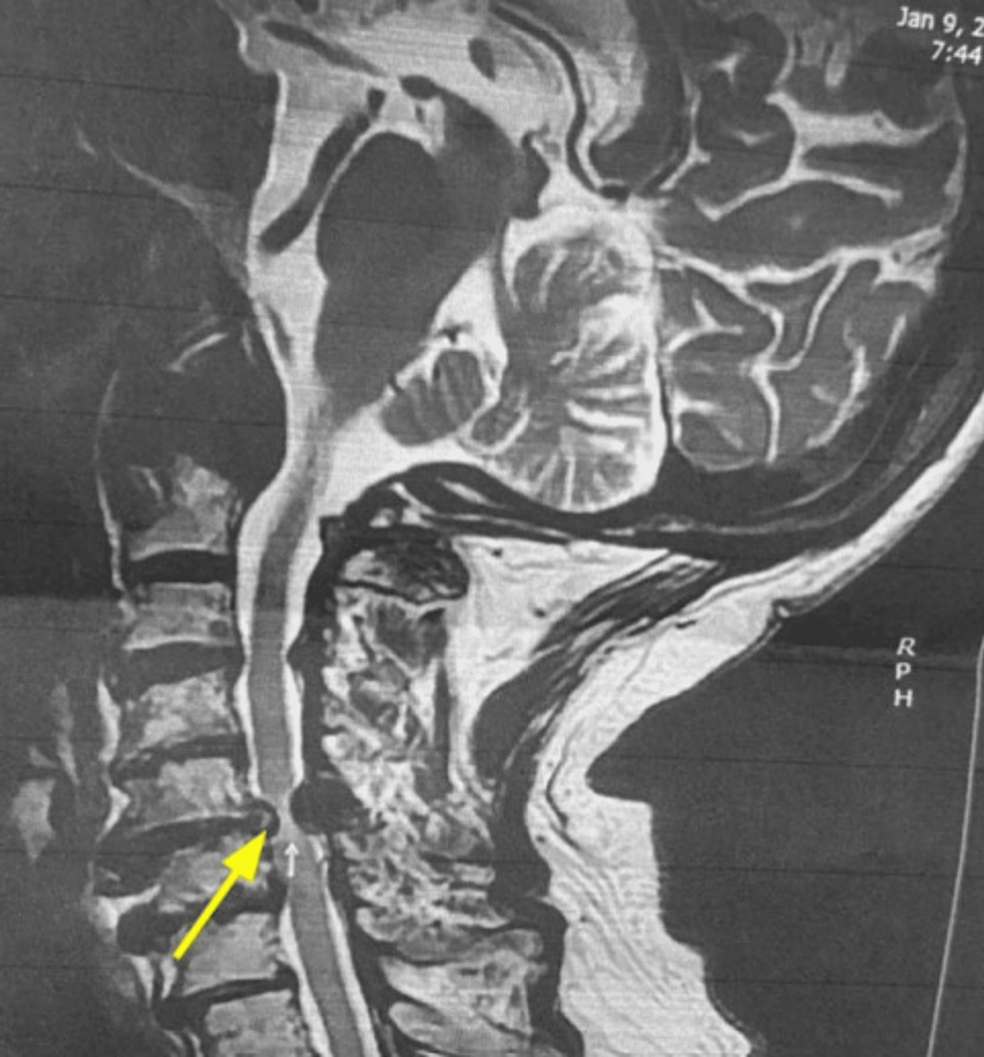
MRI of the cervical spine (axial) showing cervical stenosis, done two years prior to presentation.

A 93-year-old female with a history of atrial fibrillation, myocardial infarction status post two stents, squamous cell carcinoma (SCC) post excision, hypertension, diabetes mellitus, fibromyalgia, and hyperlipidemia, presented to the emergency room for evaluation of generalized weakness and inability to ambulate soon after a facial procedure. She was recently diagnosed with squamous cell carcinoma 15 days prior and had surgery under anesthesia for an excision on her right cheek. She did not experience any weakness after the first surgery. She underwent a second procedure, after which she lost function on the right side of her body and could not ambulate after one day. Her procedure was under procaine. The patient was able to ambulate on a walker at baseline and was able to drive before her operation. The patient had an magnetic resonance imaging (MRI) in Florida after she developed neck pain following chiropractic neck manipulation two years ago, which demonstrated cervical stenosis, and she was referred for surgical intervention (Figure 1).

**Figure 2:**
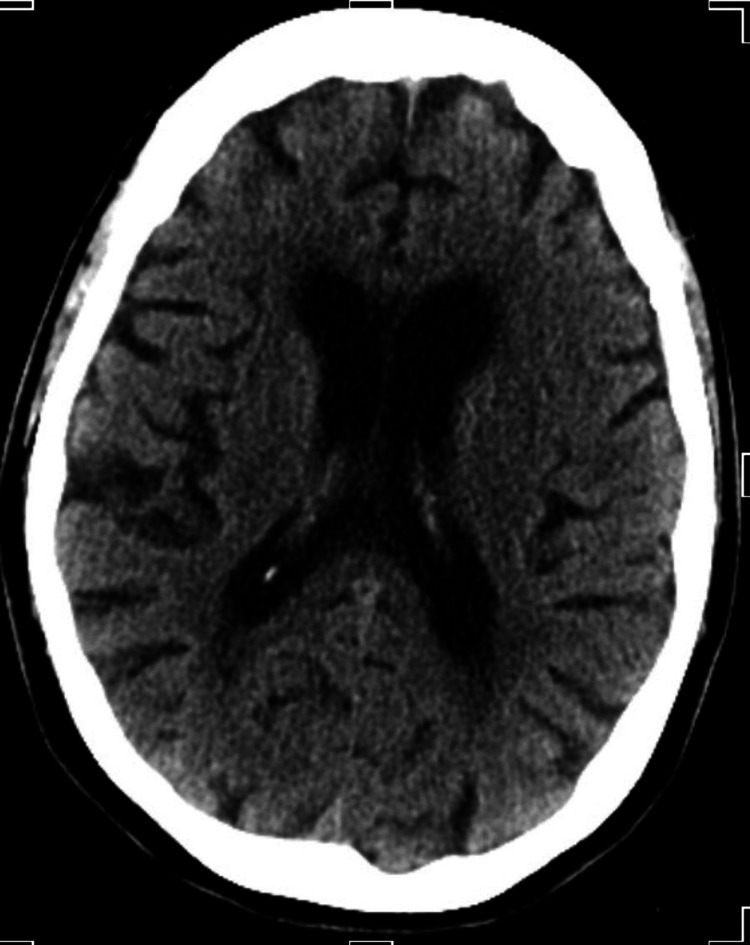
CT head without contrast showing no stroke.

The patient did not follow-up for surgery, given her age and the COVID-19 pandemic. In the ED, an x-ray of the cervical spine demonstrated spondylotic disease without discrete fracture and a computed tomography scan of the head showed no acute intracranial pathology (Figure 2).

**Figure 3:**
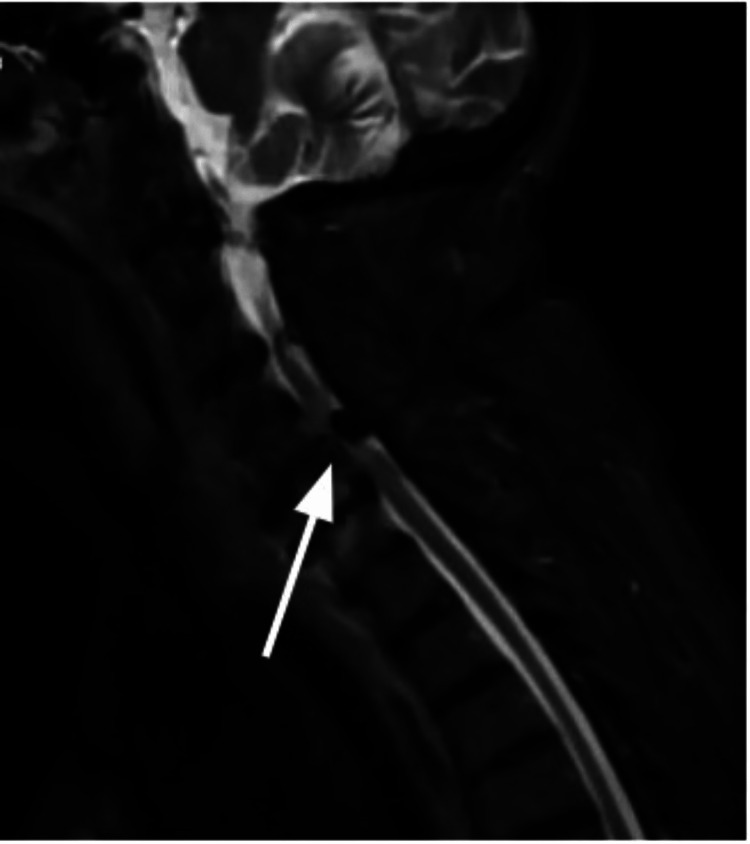
MRI cervical spine (sagittal) view of the cervical spine from admission demonstrating severe spinal canal stenosis predominantly in C5-C6 secondary to the anterolisthesis along with uncovering of the intervertebral disc and posterior facet hypertrophy. There is severe bilateral neural foraminal narrowing secondary to the anterolisthesis along with superimposed concentric disc bulge and osteophyte formation.

**Figure 4:**
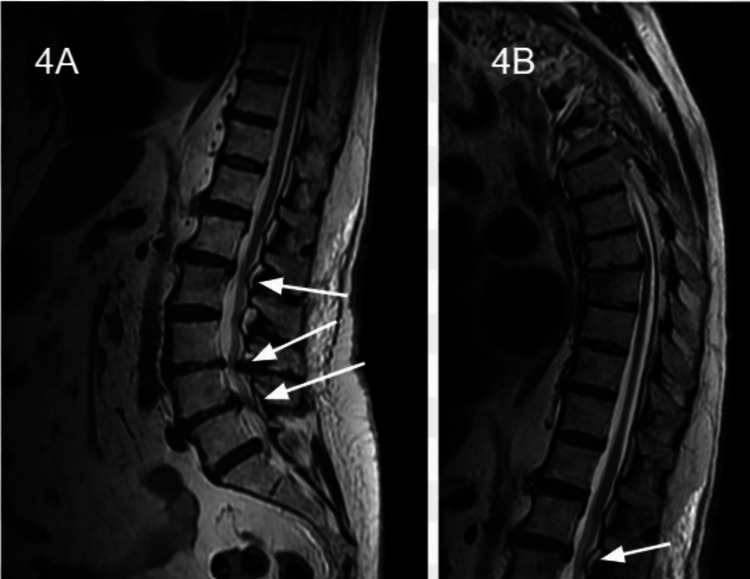
Sagittal MRI view of the thoracic and lumbar spine demonstrating spondylotic disease and disc disease causing mild cord compression at T5-T6 and T7-T8 without cord edema. No overall spinal canal or neural foraminal stenosis.

The labs were remarkable for WBC 17.6 × 10^3^/uL, blood urea nitrogen (BUN) 42 mg/dL, and Cr 1.79 mg/dL. Physical examination showed neck pain with a 7/10 intensity and generalized weakness, which was more prominent in the right upper extremity than the lower extremity. The left side was normal. The patient was given empiric antibiotic coverage of ceftriaxone and azithromycin. MRI cervical spine and thoracolumbar spine was done at admission. The cervical spine showed multi-level disc herniations and degenerative changes causing spinal canal stenosis and neural foraminal narrowing; the thoracolumbar spine also showed spondylotic disease causing mild cord compression at T5-T6 and T7-T8 without cord edema (Figures 3, 4).

She was treated with steroids and underwent cervical laminectomy and fusion. The patient’s weakness and neck pain improved significantly after the surgery, and she was discharged with a stiff neck collar, subacute rehabilitation, and activity as tolerated.

## Discussion

CSM is a degenerative change of the cervical spine that develops insidiously and impacts the spinal cord. This leads to spinal cord compression, nerve roots, and spinal nerves. Most people are diagnosed in their 50s. The classic triad of symptoms characterized by CSM is poor hand dexterity, new unsteady walking patterns, and new-onset and growing problems with motor abilities. Symptoms might not be recognized or associated with cervical spine illness in the early stages. Patients may complain of pain and paresthesia in the upper limb, nonspecific neck and shoulder pain, occipital headache, clumsiness, gait changes, and urinary retention. Physical examination may demonstrate weakness in the upper limb, and paraesthesia, including numbness and tingling in the upper limbs, may be present. Compression of the spinothalamic tract may lead to loss of proprioception, and dorsal column compression may lead to loss of pain, touch, and vibratory sensation [4]. Upper motor neuron signs like spasticity, hyperreflexia, clonus, and Babinski may be positive; the patient might have difficulty walking from toe to heel. Hoffman’s and Lhermitte’s signs are positive in some cases. Bladder involvement and urinary retention can occur. Magnetic resonance imaging (MRI) of the spine is the imaging modality of choice and is very informative and helpful in making treatment decisions. In cases where MRI is contraindicated, computed myelography is recommended, but this is an invasive process requiring an injection of dye into the spine. A compression ratio <0.4 of the spine on MRI [5] with involvement of the spinal tracts, bladder, and lower limb weakness implies a poor prognosis. Relieving spinal compression surgically leads to the resolution of many symptoms.

Most patients mention worsening symptoms present for months and attribute them to aging and corresponding functional deterioration, which often delays medical attention. Even those who may show radiographic findings may be asymptomatic clinically. The severity of CSM is classified based on gait and ambulation as in the Nurick classification [6], Ranawat classification [7] is based on pain, ambulation, and upper motor neuron signs, and the Japanese orthopedic association classification [8] is based on upper and lower limb motor and sensory functions along with bladder function. Trauma or neck manipulation may compromise an already compressed spine and lead to hemiplegia or quadriplegia, which can be confused with a stroke. The worsening of spinal cord compression after surgery in our patient may have been due to neck hyperextension sustained during anesthesia in her recent surgery. The release of spinal compression with cervical stabilization leads to rapid improvement, as seen in our patient. Considering the morbidity associated with CSM's high index of suspicion by primary care physicians, those who encounter the disease process in the early stages can prevent worsening. This case also serves as a reminder that spinal cord protective measures are necessary during surgery, especially for the elderly, as aging and degeneration of the spinal vertebra with silent CSM may be present in many.

## Conclusions

Age-related degeneration of the vertebral body, joints, and intervertebral discs leads to CSM due to compression of the cervical spinal cord or nerve. It leads to related symptoms, varying from nonspecific neck and arm pain and paraesthesia to upper motor neuron signs and hemiplegia, mimicking stroke in the elderly because of their same age. A trauma or neck manipulation may also worsen pre-existing symptoms leading to rapid worsening. Congenital spinal stenosis and osteoporosis increase the risk of CSM. MRI of the spine is the imaging modality of choice in these cases, and in patients where this is contraindicated, CT myelography is done. Surgical decompression leads to rapid relief of symptoms. A high index of suspicion may help in reducing associated morbidity. This case highlights the importance of spinal cord protective measures during surgery, especially for the elderly, as silent CSM may exist in many due to aging and degeneration.
